# Technical Feasibility of Supervision of Stretching Exercises by a Humanoid Robot Coach for Chronic Low Back Pain: The R-COOL Randomized Trial

**DOI:** 10.1155/2022/5667223

**Published:** 2022-03-09

**Authors:** Agathe Blanchard, Sao Mai Nguyen, Maxime Devanne, Mathieu Simonnet, Myriam Le Goff-Pronost, Olivier Rémy-Néris

**Affiliations:** ^1^Physical and Rehabilitation Medicine Department, Brest University Hospital, Brest, France; ^2^Brest University, Brest, France; ^3^Laboratory of Medical Imaging Processing (LaTIM), INSERM UMR 1101, Brest, France; ^4^Rambo Team, IMT Atlantique, Brest, France; ^5^Flowers Team, U2IS, ENSTA Paris, Institut Polytechnique de Paris & Inria, France; ^6^IRIMAS, Université Haute Alsace, Mulhouse, France; ^7^LEGO, IMT Atlantique, Brest, France

## Abstract

Adherence to exercise programs for chronic low back pain (CLBP) is a major issue. The R-COOL feasibility study evaluated humanoid robot supervision of exercise for CLBP. Aims are as follows: (1) compare stretching sessions between the robot and a physiotherapist (control), (2) compare clinical outcomes between groups, and (3) evaluate participant perceptions of usability and satisfaction and therapist acceptability of the robot system. Prospective, randomized, controlled, single-blind, 2-centre study comparing a 3-week (3 hours/day, 5 days/week) physical activity program. Stretching sessions (30 minutes/day) were supervised by a physiotherapist (control) or the robot. Primary outcome: daily physical activity time (adherence). Secondary outcomes: lumbar pain, disability and fear and beliefs, participant perception of usability (system usability scale) and satisfaction, and physiotherapist acceptability (technology acceptance model). Clinical outcomes were compared between groups with a Student *t*-test and perceptions with a Wilcoxon test. Data from 27 participants were analysed (*n* = 15 control and *n* = 12 robot group). Daily physical activity time did not differ between groups, but adherence declined (number of movements performed with the robot decreased from 82% in the first week to 72% in the second and 47% in the third). None of the clinical outcomes differed between groups. The median system usability scale score was lower in the robot group: 58 (IQR 11.8) points vs. 87 (IQR 9.4) in the control group at 3 weeks (*p* < 0.001). Median physiotherapist rating of the technology acceptance model was <3 points, suggesting a negative opinion of the robot. In conclusion, adherence to robot exercise reduced over time; however, lumbar pain, disability, or fear and beliefs did not differ between groups. The results of the participant questionnaires showed that they were willing to use such a system, although several technical issues suggested the KERAAL system could be improved to provide fully autonomous supervision of physical activity sessions.

## 1. Introduction

Chronic low back pain (CLBP) is a major public health issue and a leading cause of disability [1] with a large socioeconomic impact [2]. Evidence has shown that both general physical activity and a program of specific exercises alleviates both pain and disability and so improves quality of life in individuals with CLBP [3, 4]. For any exercise program to be beneficial, it needs to be performed regularly [5]. However, it is well known that long-term patient adherence to exercise programs is poor, although this is an area that has been little studied [6].

Electronic devices such as socially assistive robots can increase engagement and learning in terms of health, physical activity, and social behaviour [7, 8]. Electronic devices, known as ‘smart' devices (e.g., watches and or mobile applications), have been marketed to both the general public and to healthcare providers for patients to increase physical activity levels [9]. These devices provide reminders, exercise lists, or physiological feedback (e.g., heart rate, blood pressure, or muscle strength), however, lack the meaningful communication, explanations, and follow-up appointments that are an integral part of therapist-led rehabilitation sessions and interactions that have been shown to benefit patients with chronic low back pain [10]. Therefore, although smart devices can be programmed by clinicians to include personalized goals and allow activity monitoring for therapeutic use, at the time of writing, we are unaware of any ‘smart' system that has successfully emulated the therapist-patient interaction.

**Figure 1:**
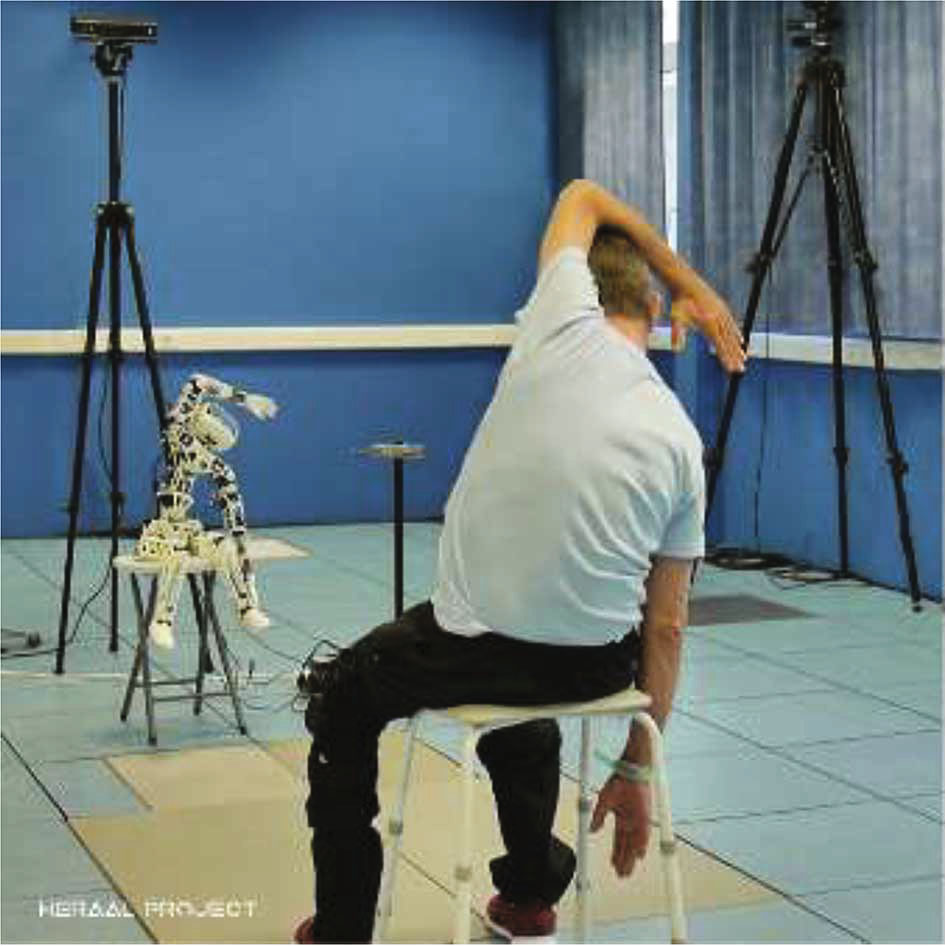
A photograph of the Poppy robot used in this study.

The aim of the KERAAL project (Kinesiotherapy and Rehabilitation for Assisted Ambient Living) is to devise a ‘smart,' humanoid robot that includes a feedback element in its rehabilitation provision to increase adherence and capitalize on the benefits of electronic devices, i.e., the robot could supervise daily, repetitive exercise sessions, and avoid daily travel to a rehabilitation centre for the patient, if provided for home-use. The overall aim of this project is to increase adherence to long-term exercise therapy for people with CLBP. The humanoid robot developed, based on the Poppy project (Figure 1), was designed to demonstrate exercises both physically (using its spine and limbs) and audibly (using a synthetic voice). In addition to providing instruction, the robot also provided correction and motivation (see Method for details) as well as recording patient performance data (e.g., the number and type of exercises performed, the number of exercises correctly performed, and the number of corrections necessary). This data could then be transmitted to clinicians to inform follow-up therapy and care. Data about the development of the Poppy robot are available at [11].

The study presented here reports the results from the first study involving the Poppy robot: the R-COOL feasibility study (RObot COach du patient chrOnique Lombalgique-robot coach for chronic low back pain). This initial study was performed in the hospital setting with the Poppy robot used to supervise a stretching session within a larger therapist-led physical activity program for CLBP.

The specific aims were (1) to compare stretching sessions performed with the Poppy robot (robot group) with those performed with a physiotherapist (control group), (2) to compare clinical outcomes between the groups, and (3) to evaluate patient perceptions of usability and satisfaction, as well as therapist acceptability of the robot system for the supervision of physical activity sessions.

## 2. Method

### 2.1. Study Design and Setting

We conducted an experimental feasibility study [12]: R-COOL was a prospective, controlled, single-blind, two-centre study conducted from October 2017 to May 2019 in two rehabilitation centres in Brittany (France). Approval was granted from the IRB of the University hospital of Brest (CPP Ouest 6), Brest, France (IDRCB No. 2017-A01097-46, 3rd October 2017). All participants signed informed consent forms for their participation. The study was registered on Clinical Trials.gov: NCT03260738, date of registration: August 24 2017.

The study was written according to the TiDier checklist (Supplementary Materials).

### 2.2. Participants

All potential participants were already enrolled in a 4-week outpatient physical activity program for people with CLBP at both sites. During the first week of the program, eligible participants were given information regarding the study. Those who provided written informed consent underwent an assessment within 2-3 days to determine if they fulfilled the inclusion criteria which were age 18 to 70 years, with nonspecific low back pain (intermittent or continuous, with or without referred pain to the posterior part of the thigh or leg) for at least 6 months. Exclusion criteria were as follows: any unstable medical conditions (acute pathology, infection, etc. that prevented inclusion in a rehabilitation program), leg pain with no lumbar pain, groin pain of any cause, and chronic diffuse pain or an inability to provide consent. The study then took place over the remaining 3 weeks of the program.

Since this was a feasibility study and there were no data in the literature relating to such an intervention on which to base a sample size calculation, the number of subjects to be included was arbitrarily fixed at 30, based on the size of the participating centres and the duration of the study.

### 2.3. Data Gathered

Baseline demographic data were gathered during the inclusion visit (day 0, D0) as well as results from the assessments usually performed on participants with CLBP in the treatment centres (see Table 1 for specific details). All data were anonymized and uploaded to the CS Randomization Clinsight software module (Ennov, San Francisco, CA, USA) which automatically and randomly allocated participants either to the control or the robot group once their details had been entered. Randomly selected block sizes of 2 or 4 were used, and investigators were blinded to the size of each block. The same clinical evaluations were performed at the end of the three-week protocol (D21). In addition, participants in both groups completed a questionnaire regarding utility and satisfaction on D2 (so that the robot group had experienced a robot-led session) and D21, and therapists completed a questionnaire about acceptability on D21. All evaluations were performed by the same doctor who was blinded to the participant's group allocation. Participating physiotherapists were all specialised in the rehabilitation of low back pain and trained in the use of the Poppy robot.

### 2.4. Primary Outcomes

The original primary outcome was the amount of daily physical activity performed by participants in each group, which we considered to represent adherence. These data were recorded by the therapists involved: each therapist noted the time spent with each participant per day. Unfortunately, the physiotherapists involved did not follow the experimental protocol instructions: when patients reported that they did not wish to perform their session with the robot, most physiotherapists provided a replacement stretching session as they found it unethical not to provide a stretching session. As a result, all participants performed the same amount of daily physical activity, and therefore, there was no difference in the mean daily physical activity time between groups.

To compensate for this, we chose the number of movements performed by the participants during each robot-led session as a proxy primary outcome measure to investigate the adherence to the robot-led sessions. The movement data were recorded automatically and very accurately by the motion capture system that was connected to the robot.

### 2.5. Secondary outcomes

#### 2.5.1. Clinical Data Recorded at D0 and D21

Change in lumbar pain was evaluated using a visual analogue scale (VAS) from 0 to 10, where 0 is no pain and 10 is the worst imaginable pain. The participant was asked to rate the maximal pain experienced in the previous 24 hours.

For change in disability, the Roland Morris (RMQ) [13] and the Dallas Pain (DPQ) [14] Questionnaires were used. The RMQ assesses the impact of low back pain on self-reported ability to perform activities of daily living using 24 questions with a final score that ranges from 0 to 24, where higher scores indicate higher levels of disability. The DPQ provided a percentage score indicating how disabling low back pain was on activities of daily living; it contained 9 questions, 3 assessed the impact on work and leisure, 3 related to anxiety/depression, and 3 measured the perceived impact on sociability.

For change in fears and beliefs (related to physical activity), the Fear-Avoidance Beliefs Questionnaire (FABQ) [15] was used to evaluate apprehension and avoidance related to physical activity. The FABQ consists of 5 questions about physical activity (scores range from 0 to 24) and 11 questions about work (scores range from 0 to 42). A higher score indicates a higher level of fears and beliefs.

Any adverse events reported by physiotherapists during each therapy session and by investigators during each evaluation visit were recorded. The investigators reported serious or nonserious research-related adverse reactions in the electronic data recording file.

#### 2.5.2. Participant Evaluation of Usability and Satisfaction

**Figure 2:**
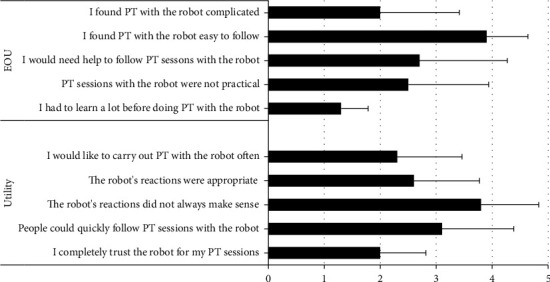
Consort flow diagram of study.

Usability was evaluated in both the robot and control groups using the System Usability Scale (SUS) [16]. This scale is composed of 10 questions: five are positively worded, and five are negatively worded (this is accounted for in the scoring method). Each answer is worth up to 10 points; scores range from 0 to 100. A score > 68 points indicates ‘above average' usability [17, 18]. Questions 1, 5, 6, 7, and 9 assess the utility of the sessions; questions 2, 3, 4, 8, and 10 assess the sessions' ease of use. The questions are shown in Figure 2.

Participant satisfaction was evaluated using a custom-made questionnaire that consisted of 2 questions, one relating to satisfaction: “I am satisfied with the care I received from my physiotherapist/Poppy robot”, and one relating to pursued intention: “I would like to continue my physiotherapy sessions with my physiotherapist / with the Poppy robot.” Responses were rated on a 5-point scale where 1 means strongly disagree and 5 means strongly agree. Space was also provided on the questionnaires for free text comments. At the end of each session, a 2-minute informal debriefing was conducted by the physiotherapist. Any comments made by the patients were recorded.

#### 2.5.3. Therapist Evaluation of Acceptability

**Figure 3:**
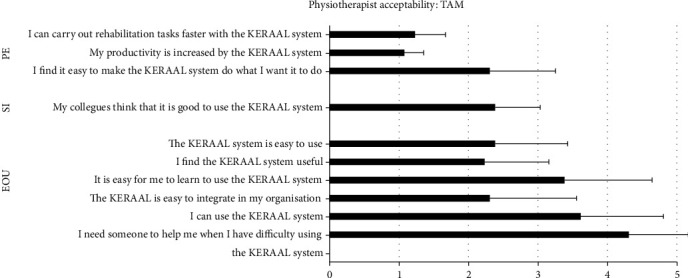
Participant usability SUS (system usability scale) questions rated by the robot group. A score of 1 = strongly disagree and a score of 5 = strongly agree, EOU: ease of use.

Acceptance of the Poppy robot by the therapists was evaluated using the unified theory of acceptance and use of technology (UTAUT) model [19] (questions relating to the technology acceptance model (TAM)). This examined three constructs: performance expectancy (PE), or the degree to which using a technology will provide benefits in performing certain activities) (3 questions, Qs 1, 2, and 6); ease of use (EOU), the degree of ease associated with the use of the technology (6 questions, Qs 3,4,5,8, and 10); and social influence (SI), which is the degree to which an individual perceives that important others (e.g., colleagues) believe he or she should use the system (1 question, Q 7). Each of the 10 questions was scored from 1 (‘do not agree') to 5 (‘strongly agree'); a mean total score of 4 or 5 thus indicated agreement. The questions are shown in Figure 3.

### 2.6. Interventions

The rehabilitation program included physiotherapy, occupational therapy, adapted sports, and hydrotherapy provided according to participants' individual needs.

The control group received conventional rehabilitation for 3 hours per day, five days a week, for the remaining 3 weeks of their program. The physiotherapy sessions included, as standard, 30 minutes of stretching. For the control group, this 30-minute standard stretching session was supervised by a physiotherapist and included stretches that targeted the trunk, neck, and lower limb muscles.

The robot group received 2.5 hours of daily conventional rehabilitation and an additional 30 minutes of stretching that was supervised by the robot, instead of a physiotherapist (see below for details of the session).

#### 2.6.1. Overview of the Poppy Robot Set-Up

The robot is an open-source, modular, and easy-to-use humanoid platform with open-source software and hardware [20], created as part of the EXPLORERS project in 2014 [21]. The Poppy (version 1.0) humanoid robot was specifically modified for the Keraal project [22] for use in a rehabilitation setting, by increasing the number and extent of available degrees of freedom as well as its social and vocal interaction. It consisted of a motorized spine with 5 degrees of freedom that allowed the performance of all exercises included in the standard stretching program [11]. The robot interacted with the participants using a preprogrammed voice synthesizer and had a display screen in the ‘head,' which showed the image of a face (Figure 1).

A 3D-motion capture camera (Xbox One Kinect sensor, Microsoft, USA) was placed 1.5 m above and slightly behind the robot, facing the participant. This camera recorded the position and orientation of the participant's body segments in real time. The data from the camera were sent to a computer for recognition and comparison of the participant's movements to an expected model for that exercise [11]. Audio feedback was then delivered to the participant via speakers in the robot's head to correct their position and provide encouragement as they performed the exercises. The robot was able to both demonstrate the exercises and to point out where mistakes were being made. During the standard 30-minute stretching session, both the participant and the robot were in a seated position, facing each other, 4 m apart.

During the first session with the robot coach, the physiotherapist spent 5 minutes explaining to the participant how the system interacts, what the robot can do and will do, and the types of instructions and feedback the robot can make, based on a prewritten guideline. At the start of each subsequent session, the therapist spent 2 minutes reminding the participant of the principles of the robot coach. The therapist then sat the robot on its chair and launched the list of exercises through the computer web interface. At the end of the session, the therapist spent around 2 minutes debriefing with the participant.

#### 2.6.2. The Robot-Mediated Stretching Sessions

The standard 30-minute stretching session consisted of (i) maximal left and right trunk rotation, (ii) left- and right-side bending (within the limits of pain), and (iii) breathing exercise, all performed in sitting. Each stretch was held for 10 seconds and was performed 10 times. The movements were performed slowly as the objective was to stretch the muscles and joints without inducing pain. The session was performed in a room where the participant was alone with the robot.

The robot was programmed to first demonstrate the movement to the participant while providing vocal explanations of what to do. The robot then instructed the participant to perform the exercises, while it continued to demonstrate them. The Kinect camera recorded the participant's movements, and these data were compared to a model of the movement that had been prerecorded by the therapists. Vocal feedback was provided by the robot after completion of each movement, using real-time information from the camera. This allowed correction of specific errors using prerecorded messages developed by physiotherapists and psychologists involved in the KERAAL project [11] such as “Your arm was not in the right position.” or “Your trunk was not flexed enough.”

Encouraging prerecorded vocal messages were also given after each exercise, e.g., “Well done!,” “You have completed all the movements.” or “One last time.”

### 2.7. Statistical Analysis

Intention-to-treat analysis was performed with an alpha risk of 0.05. In case of missing data, the analysis was based on the available data.

The original primary outcome of mean daily physical activity time was compared between groups using a Student *t*-test.

Between-group differences in changes in clinical scores (VAS, RMQ, DPQ, and FABQ scores) from D0 to D21 were evaluated using a Student *t*-test. The between-group SUS scores were compared using a Wilcoxon signed-rank test. Within group changes in SUS and satisfaction scores (D2-D21) were compared using a Wilcoxon signed-rank test.

Satisfaction scores were compared between D2 and D21 within groups and between groups at each time point using a Wilcoxon signed-rank test.

## 3. Results

**Figure 4:**
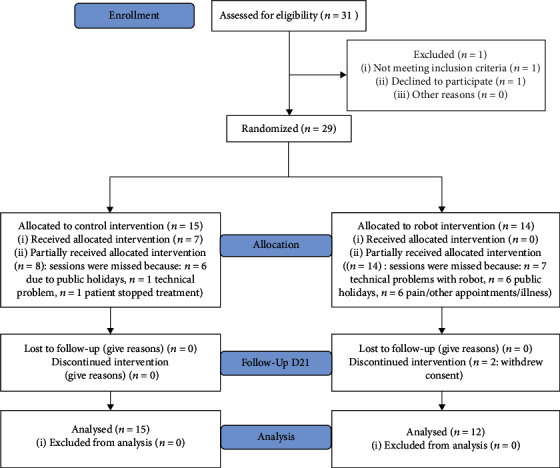
Results for each TAM (technlogy acceptance model) question rated by the physiotherapists. A score of 1 = strongly disagree and a score of 5 = strongly agree. EOU: ease of use, SI: social interaction; PE: performance expectancy.

Thirty-one participants were identified as being potentially eligible for participation (flow chart, Figure 4). Following the first assessment at D0, one patient was excluded because he was ineligible, and another did not sign the informed consent. The remaining 29 individuals were randomly allocated to either the robot or control group. After beginning the protocol, 2 participants from the robot group changed their minds about participation and withdrew consent. Thus, at D21, there were *n* = 12 participants in the robot group and *n* = 15 participants in the control group. Baseline data are shown in Table 1.

### 3.1. Primary Outcome

The mean number of days of attendance at the rehabilitation centre was similar between the groups (robot group: 13.8/15, control group: 13.9/15). The reasons for failure to attend were mostly due to pain that prevented participation. As expected, there was no statistically significant difference in the between-group mean daily physical activity time (robot = 228.8 minutes (CI: 190.7-266.9); control = 226.5 minutes (CI: 190-262.9), *p* = 0.92). This was because the physiotherapists replaced any missing robot-led sessions with a therapist-led stretching session, and in consequence, both groups of participants received the same amount of therapy. The mean number of robot-supervised sessions was 10.6/15 due to robot malfunction.

A difference was observed in the proxy primary outcome: the proportion of movements performed by the participants in the robot-led sessions. The total proportion of movements performed with the robot decreased from 82% in the first week to 72% in the second and 47% in the third.

### 3.2. Secondary Outcomes

#### 3.2.1. Clinical

There were no between-group differences in the changes recorded between D0 and D21 in any of the clinical outcomes (Table 2).

#### 3.2.2. Participant Evaluation of Usability (SUS) and Satisfaction

The SUS score was significantly lower in the robot group than the control group at both D2 and D21. There was no within-group difference in SUS scores from D2 to D21 in either group (Table 3).

Satisfaction was significantly lower in the robot group than the control group at both D2 and D21. There was no within-group change in satisfaction score from D2 to D21 in either group (Table 3). The results for each question of the SUS are shown in Figure 2.

The pursue intention score was significantly lower in the robot group than the control group at both D2 and D21. There was no within-group difference in pursued intention score from D2 to D21 in either group, although there was a tendency towards a drop in score in the robot group (Table 3).

#### 3.2.3. Therapist Evaluation of Acceptability (TAM)

Thirteen physiotherapists who were all involved in setting up the robot stretching sessions completed the questionnaire. The median score for questions assessing performance expectancy was 1.6/5 (IQR = 0.267) and for ease of use was 3/5 (IQR = 0.298). The robot was therefore perceived as easy to use, but not very useful. The median score for the social factor questions was 2/5 (IQR = 0.393), indicating that the physiotherapists had a negative opinion about the robot. The results for each question of the TAM are shown in Figure 3.

### 3.3. Adverse Events

Nine adverse events, 3 of which were not related to the study, and 6 of which were musculoskeletal (acute LBP, tension-type headache, cervical and scapular pain, and pain in the sciatic distribution) were reported by robot group participants. Four adverse events were reported by the control group participants, 1 of which was not related to the study, and 3 of which were musculoskeletal (acute LBP).

## 4. Discussion

The work presented in this paper evaluated the feasibility of using a humanoid robot to deliver stretching exercise therapy for individuals with CLBP. For practical reasons, this initial study was performed in the hospital setting; however, the long-term aim of the project is to use the robot to supervise and promote adherence to a home exercise program. Participation in sessions with the Poppy robot declined over the three weeks of the intervention, showing reduced adherence. Importantly, there were no differences in the number of side effects reported by the participants of either group, and no serious adverse events occurred, showing that the robot-led sessions were safe. There were no between-group differences in change in the clinical outcomes between D0 and D21. Participants in the robot group were less satisfied with the robot supervision than those in the control group who were supervised by physiotherapists, and 2 participants declined further participation after 1 week of working with the device. The physiotherapist rating of the system acceptability was low. The main outcome of the study was therefore that the KERRAL system is not ready to be used for rehabilitation.

### 4.1. Participant Point of View

Recent reviews into the effects of electronic feedback systems in CLBP have not shown that these devices are more effective than minimal interventions (e.g., waiting list or brief education) or conventional rehabilitation in either reducing reported pain and disability or in increasing long-term physical activity levels [23, 24]. Feedback systems like pedometers [25] and website or smartphone applications that encourage physical activity, often with interactive elements like a logbook, individualized information, small fun exercises like quizzes, personalized objectives, motivational feedback, and forums have been tested with no consistent long-term benefits over conventional rehabilitation [26].

Relational robots, like the Poppy robot, that give encouraging messages and provide appropriate facial expressions (on a screen ‘face') which are adapted to each piece of feedback have been found to be better accepted by humans than nonrelational robots that only give exercise instructions [27]. Although participants were globally dissatisfied with the Poppy robot, this was mostly due to technical issues. The results of the pursue intention question showed that at the start of the therapy (D2) they were willing to use the system. The results of the SUS questionnaire showed that participants found sessions with the Poppy robot somewhat complicated, not always easy to follow, and that the robot's reactions were not always appropriate. The comments left by a few participants on the questionnaire indicated that several liked the idea of using the robot; however, the technical difficulties constituted a barrier: “Problems because of the Poppy robot dysfunction, otherwise very good” (participant 15). Some participants complained to their therapists that the exercises were repetitive and boring during the after-session debriefings.

We believe that this was due to several issues: firstly, inadequacies in the motion-capture system resulted in inappropriate feedback. We chose the Kinect system because it is low-cost and therefore feasible for self-rehabilitation in patients' homes in the long-term. We believed that the system would be sufficiently accurate in view of the simplicity of the exercises developed for the protocol. However, subsequent analysis of the videos revealed that axial rotation and lateral trunk flexion are under evaluated by the Kinect system. These incorrect instructions could therefore be explained by the limits in motion detection inherent to the Kinect motion capture camera [28, 29]. This likely led to a lack of trust in the Poppy robot, a factor that is essential for the success of such robots [30], as demonstrated in the following comment “explanations needed for the robot's movements–a human presence is essential” (participant 12). Secondly, the comments made by the physiotherapists involved suggested the standardized exercises were boring, especially in comparison to the range and diversity of exercises provided by an experienced physiotherapist; the ability to adapt exercises to the participants is a key feature of efficient rehabilitation [30, 31]. A greater diversity would probably have reduced the boredom.

Feedback on performance (position, intensity, and duration) was found to be an important criterion for individuals with CLBP to use technological systems to guide exercises [32]. Exercises should also be patient specific to aid adherence [33]. It has also been reported that, over time, a lack of coaching is an important factor in the gradual loss of interest in the physical activity [3]. A review of qualitative studies found that patient engagement in exercise required the level of difficulty to be appropriate and the exercises to be personalised and supervised with feedback and correction and to be fun [10]. These dimensions were lacking in the exercises proposed by the Poppy robot.

Comments such as “I consider that Poppy is a good complement between two sessions with a physiotherapist” (participant 9) are positive for the long-term aim of this project, which is not to substitute sessions with a physiotherapist but to provide a means of motivation and coaching for patients to perform home exercise programs.

### 4.2. Physiotherapist Point of View

The physiotherapists generally considered that the Poppy robot was not useful, although it appeared to be neither easy nor difficult to use (majority of ‘I don't know' responses). This was also demonstrated by the fact that, rather than encouraging participants to carry out their sessions with the Poppy robot as they had been instructed, they replaced missed sessions with in-person sessions.

In general, physiotherapists have been found to have positive attitudes towards the use of robotic technology in rehabilitation, as long as they fulfil their intended goals, are not time consuming to set up, and therapists receive adequate training in their use [34, 35]. Negative reactions may occur if therapists perceive an increase in their workload [36]—the results of the UTAUT-TAM questionnaire showed that the majority of physiotherapists replied ‘totally disagree' to the questions regarding work and productivity, suggesting that use of the Poppy robot generated more work for them, rather than relieving them. Performance expectancy has been found to be a powerful factor influencing therapist's willingness to use a technological device [34]. The technological issues surrounding the robot system meant that it did not fulfil performance expectancy, either in improving patient outcomes or facilitating their job performance.

### 4.3. Perspectives: Future Directions

The essential messages learned from this study are that a robotic system designed for rehabilitation must provide appropriate feedback that participants perceive to be reliable and correct and that the exercises must be varied. In the light of these results, we are currently working to improve the accuracy of recording of the Poppy robot. We are also developing a greater range of exercises as well as improving feedback quality using more powerful movement detection and classification algorithms. For the robot feedback to be appropriate, it must be individualised: for example, patients of different ages, pain levels, etc. require different ranges of motion. It must therefore be possible for the therapist to calibrate the system for the individual.

A variety of robotic devices are now available for rehabilitation, and many rehabilitation centres include upper and/or lower limb robotic therapy in their rehabilitation programs. Commercial and organizational processes have therefore been established for the distribution of robots in the hospital setting. However, commercial networks will need to be developed for the distribution of humanoid robots to be used in the home setting. Pathways like those already used for electronic wheelchairs and similar devices could be adapted for this purpose. With regard to costs, medical-economic studies of existing rehabilitation robotic devices have found better economic outcomes for robotic than conventional therapy [37]. A remaining issue is that our study was based on an open-source robot https://www.poppy-project.org/fr/; a commercial version of such an application should be based on a more robust industrial robot. However, most industrial humanoid robots (such as Nao by Aldebaran Robotics [38]) do not have as many degrees of freedom as the Poppy robot, particularly at the spine. This issue must be resolved before transfer to an industrial product and a commercial application can be considered.

Few studies in the literature have described the use of robots as a support for self-rehabilitation. Current descriptions are mostly limited to pilot tests published in conference proceedings [24, 38–40]. We believe that such systems have real potential to increase adherence to long-term exercise programs for chronic conditions; however, much work is needed to make this happen.

### 4.4. Study Limitations

The main limitation of this study is that the robot system was not technically ready for use in the rehabilitation setting, and the study was perturbed by the robot malfunction. Additionally, the physiotherapists replaced missed robot sessions with in-person sessions, preventing between-group comparisons for the primary outcome. Another limitation is that the SUS and UTAUT-TAM are not specific to health systems and were adapted for the study. Thirty patients were recruited into the study over a period of 2 years. This slow rate was due to the strict inclusion criteria.

## 5. Conclusion

This study showed that the use of the Poppy robot is safe. Adherence to robot sessions decreased over the study duration, suggesting that in its current state it is not feasible for the supervision of physical activity sessions; however, there was no negative impact on clinical outcomes. The results of the participant questionnaires suggested that they were willing to use the system but were discouraged by the technical issues that occurred. The data from this study have been used to improve the system and further studies will be undertaken.

## Figures and Tables

**Table 1:** Demographic and baseline participant data.

	Robot group	Control group
	*n* = 13	*n* = 15
Sex (M : F)	1 : 01	3 : 02
	Mean ± SD	Mean ± SD
Age	42.9 ± 12.8	43.8 ± 9.7
BMI	27.6 ± 5.5	25.2 ± 5.0
VAS during the previous 24 hours	6.1 ± 2.2	6.5 ± 1.9
RMQ	11.8 ± 3.7	10.4 ± 3.5
DPQ-daily activity	70.6 ± 13.0	63.0 ± 14.8
DPQ-work/leisure	51.4 ± 19.0	46.4 ± 17.2
DPQ-anxiety/depression	35.0 ± 19.4	33.6 ± 14.2
DPQ-sociability	31.4 ± 22.7	27.1 ± 19.8
FABQ-physical activity	14.4 ± 7.3	14.3 ± 5.7
FABQ-work	31.8 ± 12.2	23.3 ± 13.1
Duration of LBP (months)	66.4 ± 82.9	113.4 ± 143.4
	*n*	*n*
Acute LBP = 1 to 3 events	0 (0%)	2 (13%)
Acute LBP > 3 events	7 (50%)	6 (40%)
Back surgery	2 (14%)	2 (13%)
Professional activity during the previous 12 months	6 (43%)	11 (73%)
Work interruption due to LBP	7 (88%)	3 (75%)

M: male; F: female; BMI: body mass index; VAS: visual analogue scale; RMQ: Rolland-Morris Questionnaire; DPQ: Dallas Pain Questionnaire; FABQ: Fear-Avoidance Beliefs Questionnaire, LBP: low back pain.

**Table 2:** Changes in clinical scores from D0 to D21.

	Change from D0-D21		
	Control	Robot	*p* value
	*n* = 15	*n* = 12	
VAS previous 24 hours	−1.9 ± 2.4	−1.6 ± 2.5	0.69
RMQ	−1.1 ± 3.6	−2.1 ± 2.5	0.47
DPQ-daily activity	−4.0 ± 10.9	−12.90 ± 13.4	0.10
DPQ-work/leisure	−7.3 ± 21.0	−5.0 ± 11.1	0.76
DPQ-anxiety/depression	−9.6 ± 14.9	−1.0 ± 12.1	0.14
DPQ-sociability	−3.2 ± 15.5	2.50 ± 8.6	0.30
FABQ-physical activity	−2.4 ± 6.3	−2.6 ± 5.0	0.94
FABQ-work	−2.0 ± 6.8	−3.0 ± 4.1	0.67

Values presented are means ± SD. VAS: visual analogue scale; RMQ: Rolland-Morris Questionnaire; DPQ: Dallas Pain Questionnaire; FABQ: Fear-Avoidance Beliefs Questionnaire; LBP: low back pain.

**Table 3:** Within- and between-group comparisons of utility and satisfaction evaluated by the participants.

		Robot	Control	Between-group
				*p* value
SUS	D2	64 (9.5)	85 (5.4)	0.007
	D21	58 (11.8)	87 (9.4)	<0.001
Within-group *p* value		0.22	0.19	
Satisfaction	D2	3.5 (0.6)	5 (0.3)	<0.001
	D21	2.5 (0.8)	5 (0.4)	<0.001
Within-group *p* value		0.19	0.83	
Pursue intention	D2	4 (0.8)	5 (0.4)	0.01
	D21	2 (0.8)	4 (0.3)	<0.001
Within-group *p* value		0.063	0.74	

Values presented are medians (IQR). SUS: system usability scale. Total SUS scores are calculated out of 100 following a specific method [16] that accounts for positively and negatively worded questions. A score > 68 points indicates ‘above average' usability [17, 18].

## Data Availability

The datasets used and/or analysed during the current study are available from the corresponding author on reasonable request.

## Supplementary Materials

Supplementary Materials The TIDieR (Template for Intervention Description and Replication) Checklist^∗^: information to include when describing an intervention and the location of the information.
